# Looking Back and Looking Forward

**Published:** 2007-12-15

**Authors:** Jaya K Rao

**Affiliations:** Healthy Aging Program, Centers for Disease Control and Prevention

Consider the world of the 1950s. Ten percent of American households had a television, which displayed only in black and white. Telephones had rotary dials and party lines, and they were used only for conversation. The average life expectancy at birth was 69 years (1). Massive randomized clinical trials were in progress to test a new vaccine for polio (2) and streptomycin and isoniazid treatments for tuberculosis (3). Heart disease and stroke were gaining recognition as the leading noninfectious causes of death in the United States. Dwight D. Eisenhower was the President. And, were it not for President Eisenhower, I might not have had the opportunity to write this editorial for *Preventing Chronic Disease*.

I am the second child of two remarkable people who came to the United States many years ago. In the early 1950s, as a newly trained and highly skilled biochemist, my father was recruited to be a member of the antibiotic discovery group of Chas. Pfizer & Co., Inc. Because he was a scientist with the specialized skills necessary to fulfill an urgent national need, his application for permanent U.S. residency was rapidly approved. Confident that his wife's and child's applications would also be approved as his dependents, my father left India in 1954 to begin his new job.

At the time, a quota system severely restricted the number of immigrants into the United States from non-Northern European countries. Obtaining an immigrant visa involved a mountain of paperwork and numerous clearances. While examining my mother for the medical clearance, the physician in Bombay noticed her slender build and suspected a parasitic infection as the underlying cause. An extensive evaluation ensued, which involved tests for various communicable diseases, including one for tuberculosis (the results were negative) and a prolonged course of empiric treatment for possible dysentery.

Obtaining the medical clearance for my mother took a year. By the time she and my sister were approved to travel, their authorization for immigrant visas had expired. With the immigrant quota for South Asian applicants filled for more than 10 years into the future, it appeared unlikely they would ever be able to join my father. Understandably, my father told his supervisor that he planned to return to India. Concerned about losing a valuable employee, Pfizer attorneys contacted Senator Herbert Lehman (D-NY) for assistance. On August 1, 1956, Senator Lehman introduced private legislation (4) which was passed by both houses of Congress and signed by President Eisenhower granting special permission for my mother and sister to immigrate into the United States. They arrived before the year's end.

The 1950s is not only an important time in my family's history but is also a period of key advances in population health. By the middle of the 20th century, the public health community had become interested in collecting data on possible risk factors related to chronic diseases. In 1956, President Eisenhower signed the National Health Survey Act, authorizing a continuing survey "to secure accurate and current statistical information on the amount, distribution, and effects of illness and disability in the United States" (5). As a consequence, the National Household Health Interview Survey (1957) and the National Health Examination Survey (1960) were created. Now known as the National Health Interview Survey and the National Health and Nutrition Examination Survey, respectively, these surveillance systems, along with research from the Framingham cohort study (1948), produced data that advanced our understanding of the risk factors for cardiovascular disease, cancer, and other chronic conditions; they also led to the development of public health interventions and new medical treatments.

Since the 1950s, the average life expectancy at birth has increased from 69 to 78 years (1). Today, people 65 years of age can expect to live an additional 18.7 years, or 5 more years than their counterparts during the 1950s (1). The 1950s also were the early days of the baby boom generation, a group that will contribute substantially to the growth of the aging population. By 2030, 20% of the entire U.S. population will be adults 65 years or older (6). As people live longer, their expectations regarding quality of life throughout the lifespan are changing. And, although chronic diseases such as cardiovascular disease, cancer, and diabetes remain important public health concerns as major causes of illness, disability, and mortality among adults older than 65, we are beginning to see a greater focus than heretofore on other health and lifespan issues, including cognitive and emotional health (7,8), caregiving (9), and end-of-life issues (10).

In this issue of *Preventing Chronic Disease*, we highlight emerging topics related to the health of older adults (i.e., adults aged 50 years or older). We are honored to include former First Lady Rosalynn Carter's editorial, which focuses on her important work related to caregiving (11). Chapman and Perry (12) and Snowden et al (13) focus on depression among older adults. Glass and Nahapetyan (14) analyzed qualitative data to describe the perspectives of baby boomers and older adults on planning for the end of life. Mayer et al (15), Batik et al (16), and Nguyen et al (17) focus on efforts to promote and measure physical activity among community-dwelling older adults. Shenson and colleagues (18) describe their experiences adapting a preventive service approach for older adults that was successful in New England communities for a community in the U.S. Southeast. Because data indicate that limited health literacy is an important problem among older adults, Friedman and Kao (19) assessed the reading level and cultural appropriateness of Web sites containing information about prostate cancer. Finally, Aldrich and Benson (20) discuss the practical aspects of emergency preparedness as it applies to older adults.

December 10, 2006, marked the 50th anniversary of my mother's arrival in the United States. Like any family, mine experienced many changes during these 5 decades. My parents went on to have two more children, my brother and me, and to build their life in the United States. My mother transformed herself from a quiet young woman from a small village in south India who spoke little English to a full-fledged American citizen who speaks fluent English and enjoys discussing domestic and international issues with family and friends. Over time, my mother has changed from someone who was the major family caregiver to a woman who accepts modest assistance from her children. Although she moves slowly now because of severe arthritis, she has the youngest spirit of anyone I know.

Because of improvements in population health, my siblings and I have had more quality time with our parents than they had with their parents. I suspect that we are not alone in this regard. We should consider the growth of the aging population a public health triumph. At the same time, we must also recognize that addressing the needs of a sizeable population of older adults with chronic disease will be a challenge for public health. Time will tell how well we meet this challenge.
